# *Teredinibacter waterburyi* sp. nov., a marine, cellulolytic endosymbiotic bacterium isolated from the gills of the wood-boring mollusc *Bankia setacea* (Bivalvia: Teredinidae) and emended description of the genus *Teredinibacter*

**DOI:** 10.1099/ijsem.0.004049

**Published:** 2020-02-20

**Authors:** Marvin A. Altamia, J. Reuben Shipway, David Stein, Meghan A. Betcher, Jennifer M. Fung, Guillaume Jospin, Jonathan Eisen, Margo G. Haygood, Daniel L. Distel

**Affiliations:** ^1^​ Ocean Genome Legacy Center, Northeastern University, Nahant, MA, USA; ^2^​ School of Biological Sciences, University of Portsmouth, Portsmouth, UK; ^3^​ Downstream Strategies, Alderson, WV, USA; ^4^​ Mendel Biotechnology, Hayworth, CA, USA; ^5^​ College of Biological Sciences, University of California, Davis, CA, USA; ^6^​ Department of Medicinal Chemistry, College of Pharmacy, University of Utah, UT, USA

**Keywords:** cellulolytic symbiont, *Teredinibacter*, bivalve, Teredinidae, shipworm, *Bankia setacea*

## Abstract

A cellulolytic, aerobic, gammaproteobacterium, designated strain Bs02^T^, was isolated from the gills of a marine wood-boring mollusc, *Bankia setacea* (Bivalvia: Teredinidae). The cells are Gram-stain-negative, slightly curved motile rods (2–5×0.4–0.6 µm) that bear a single polar flagellum and are capable of heterotrophic growth in a simple mineral medium supplemented with cellulose as a sole source of carbon and energy. Cellulose, carboxymethylcellulose, xylan, cellobiose and a variety of sugars also support growth. Strain Bs02^T^ requires combined nitrogen for growth. Temperature, pH and salinity optima (range) for growth were 20 °C (range, 10–30 °C), 8.0 (pH 6.5–8.5) and 0.5 M NaCl (range, 0.0–0.8 M), respectively when grown on 0.5 % (w/v) galactose. Strain Bs02^T^ does not require magnesium and calcium ion concentrations reflecting the proportions found in seawater. The genome size is approximately 4.03 Mbp and the DNA G+C content of the genome is 47.8 mol%. Phylogenetic analyses based on 16S rRNA gene sequences, and on conserved protein-coding sequences, show that strain Bs02^T^ forms a well-supported clade with *Teredinibacter turnerae *. Average nucleotide identity and percentage of conserved proteins differentiate strain Bs02^T^ from *Teredinibacter turnerae* at threshold values exceeding those proposed to distinguish bacterial species but not genera. These results indicate that strain Bs02^T^ represents a novel species in the previously monotypic genus *Teredinibacter* for which the name *Teredinibacter waterburyi* sp. nov. is proposed. The strain has been deposited under accession numbers ATCC TSD-120^T^ and KCTC 62963^T^.

The genus *Teredinibacter* was erected in 2002 to accommodate a single valid published species, *Teredinibacter turnerae *, represented by 58 similar strains of *Gammaproteobacteria* isolated from the gills of 24 diverse species of the wood-boring and wood-feeding bivalve family Teredinidae, commonly known as shipworms [1]. The genus remains monospecific to date. Strains of *T. turnerae* are cellulolytic, microaerobic, heterotrophic, diazotrophic, motile, slightly curved rods that bear a single polar flagellum when grown in culture. *T. turnerae* is unusual among intracellular endosymbionts of marine taxa in that it is among the few that have been brought into pure culture. To our knowledge, *T. turnerae* is the only cellulolytic intracellular endosymbiont formally described to date, although a chemoautotrophic sulfur-oxidizing symbiont, *Thiosocius teredinicola *, from the gills of the giant sediment-dwelling shipworm *Kuphus polythalamius* has recently been described [2].

Shipworms and their symbionts are of considerable ecological and economic importance [3]. As the principal consumers of wood in brackish and marine ecosystems [4], shipworms play an important role in coastal marine carbon cycles [5, 6]. The burrowing and feeding activity of these worm-like bivalves also cause extensive damage to coastal wooden structures, from boats, jetties and piers, to fishing and aquaculture equipment, resulting in billions of dollars of damages around the world each year [3]. Like many animals capable of digesting lignocellulose in woody or leafy plant material, shipworms rely on enzymes produced by symbiotic microorganisms to aid in the breakdown of this recalcitrant material [7]. However, unlike these other animals, shipworms harbour bacterial symbionts intracellularly in a specialized tissue within their gills [8] but lack bacteria in the portion of the gut responsible for wood digestion [9]. Recently, it has been demonstrated that the intracellular symbiont community in the gills of the shipworm *Bankia setacea* secrete cellulolytic enzymes that are selectively transported from the gill to gut, a digestive strategy which is thought to be unique to shipworms [10]. In that study, four gammaproteobacterial endosymbionts, designated as strains Bs02^T^, Bs08, Bs12 and Bsc2, were isolated, grown in pure culture and their genomes sequenced. The genomes of these four symbiont strains were shown to account for the majority of genes detected in the gill metagenome of *B. setacea* indicating that these bacteria constitute the dominant components of *B. setacea* gill symbiont community [10]. Here, we characterize one of these four strains, Bs02^T^, for which the name *Teredinibacter waterburyi* sp. nov. is proposed. This strain is deposited to the American Type Culture Collection and Korean Collection for Type Cultures under the accession numbers ATCC TSD-120^T^ and KCTC 62963^T^, respectively.

Strain Bs02^T^ was isolated in pure culture from the gills of the shipworm *Bankia setacea* as described by O’Connor *et al.* [10]. Briefly, approximately 100 mg fresh gill tissue was homogenized by hand in 1 ml ice-cold sterile seawater buffered with 50 mM HEPES, pH 8.0 in a sterile glass Dounce homogenizer. Homogenate was streaked on 1.0 % Bacto agar shipworm basal medium (SBM) plates, supplemented with 0.2 % w/v powdered cellulose (Sigmacell Type 101; Sigma-Aldrich) and 0.025 % NH_4_Cl and incubated at 30 °C. After 20 days, colonies were selected and subjected to multiple rounds of re-streaking on fresh plates until single monoclonal colonies of uniform appearance were obtained. For all subsequent experiments the strain was propagated in test tubes (18 mm × 150 mm) containing 6 ml liquid SBM with either 0.2 % cellulose or 0.5 % galactose (w/v) added as the as sole carbon source at 20 °C with shaking at 100 rpm, unless specified otherwise. Frozen stocks were prepared from 7 to 10 day old cultures as follows: 3 ml of turbid liquid culture were pelleted by centrifugation at 5000 *g* at 20 °C for 5 min; the supernatant was removed, replaced with 0.5 ml of fresh SBM liquid medium, and gently mixed to disperse the cells; 500 µl of the cell suspension were transferred to a sterile cryotube containing 35 µl filter-sterilized DMSO and frozen at −80 °C. Frozen stocks were revived by thawing the entire vial and using 200 µl to inoculate and grow a 6 ml liquid culture in SBM cellulose medium as described above.

Cells of strain Bs02^T^ were transferred to SBM liquid medium and SBM agar plates supplemented with a variety of carbon sources with and without 0.025 % NH_4_Cl (w/v) or 5 mM NaNO_3_ added as a nitrogen source. Growth was observed with cellulose (0.2 %), carboxymethylcellulose (0.5 %), cellobiose (0.5 %), xylan (0.5 %), sucrose (0.5 %), xylose (0.5 %) arabinose, galactose (0.5 %) or glucose (0.5 %) but not without NH_4_Cl, indicating that strain Bs02^T^ does not have the capability to fix atmospheric dinitrogen like *T. turnerae *. Growth was also observed on media containing cellulose or galactose supplemented with NaNO_3_. No growth was observed on formate, acetate or propionate (all at 0.1 % w/v) with or without the addition 0.025 % NH_4_Cl. The capacity of strain Bs02^T^ to anaerobically respire nitrate was tested by placing inoculated SBM agar plates supplemented with 0.2 % cellulose and 5 mM NaNO_3_ inside an anaerobic pouch (BD GasPak EZ Anaerobic Pouch). No growth was detected even upon prolonged incubation.

To maintain batch-to-batch consistency during growth optima measurements in SBM liquid medium supplemented with 0.5 % galactose (w/v) and 0.025 % NH_4_Cl, the natural seawater component of the medium was replaced with a chemically-defined seawater substitute containing (l^−1^ distilled water) 23.926 g NaCl, 4.008 g Na_2_SO_4_, 0.667 g KCl, 0.196 g NaHCO_3_, 0.098 g KBr, 0.026 g H_3_BO_3_, 0.003 g NaF, 10.831 g MgCl_2_·6H_2_O, 1.518 g CaCl_2_·2H_2_O and 0.024 g SrCl_2_·6H_2_O. Optimal growth pH in liquid culture was estimated by observing growth rate at intervals of 0.5 units from pH 5.5 to pH 10.0, using media buffered with 20 mM Good’s Buffer with MES for pH 5.5–6.5, HEPES for pH 7.0–8.0, TAPS for pH 8.5 and CHES for pH 9.0–10.0. Optimum salinity for growth was estimated by adjusting the NaCl concentrations of the medium from 0.0 M to 1.0 M measured at intervals of 0.1 M. Temperature optima were estimated by observing growth rate at temperatures from 5 °C to 35 °C measured at 5 °C intervals. Specific growth rates (µ) were estimated using the exponential curve-fitting function in Microsoft Excel and absorbance values measured at 600 nm at 90 min intervals during exponential growth. Temperature, pH and salinity optima and ranges for growth were 20 °C, (range 10–30 °C), 8.0 (pH 6.5–8.5) and 0.5 M NaCl (range 0.0–0.8 M), respectively, when grown in SBM supplemented with galactose and NH_4_Cl. Under these conditions, the specific growth rate was 0.096 hr^−1^ (doubling time 7.2 hr). Unlike most marine bacteria, growth of strain Bs02^T^ does not require concentrations of Ca^+2^ and Mg^+2^ ions similar to those found in seawater (typically ∼10 mM Ca and 50 mM Mg). Instead, growth was observed on modified SBM liquid medium containing 0.5–10 mM CaCl_2_·2H_2_O and 0.05–50 mM MgCl_2_·6H_2_O.

When grown on SBM plates supplemented with 0.2 % cellulose, strain Bs02^T^ exhibits minute colonies that are initially slightly raised, circular, shiny and translucent. As colonies mature, growth occurs below the agar surface forming an inverted dome shape with depth similar to width. Mature colonies are off-white to beige in colour and are surrounded by a halo of clearing indicating hydrolysis of the cellulose. Cell morphology was examined using phase contrast light microscopy (Nikon Eclipse Ni-U and Nikon NIS Elements) and transmission electron microscopy (TEM). When grown in liquid media as described above, cells are initially motile and unattached, but form aggregates that may condense into biofilms as the density of the culture increases. Cells may also attach to insoluble substrates such as cellulose fibres during growth, forming sheet-like matrices. Cells are straight to slightly curved motile rods that are 2–5 µm long and 0.4–0.6 µm wide. Gram stain type was determined using a Gram Staining Kit (Sigma-Aldrich) following the manufacturer’s suggested protocol. Cells of strain Bs02^T^ are Gram-stain-negative. Cells for TEM were grown in 5 ml of liquid SBM supplemented with 0.5 % galactose (w/v) and were pelleted by centrifugation for 10 min at 8000 *g*. Resulting bacterial pellets were fixed for 2 h using 2.5 % glutaraldehyde in a 0.1 M sodium cacodylate (pH 7.2), followed by 2×15 min washes in a 0.1 M sodium cacodylate at 4 °C. Samples were post-fixed for 2 h in 1 % osmium tetroxide in 0.1 M sodium cacodylate, followed by 2×15 min washes in a 0.1 M sodium cacodylate at 4 °C. Samples were then dehydrated through an ethanol series (30, 50, 70, 85 and 95 % ethanol for 15 min at each stage), with a final dehydration stage in absolute ethanol for 1 h. Dehydrated samples were embedded in Spurr resin overnight then polymerised by heating to 60 °C for 24 h. Embedded samples were sectioned (50–200 nm thickness) using the Reichert Ultracut E (Leica), mounted on TEM square mesh copper grids and stained with 5 % uranyl acetate and Reynolds lead citrate for approximately 10–15 min each. Resulting samples were visualised on the JEOL JEM 1010 Transmission Electron Microscope. Flagella were observed by negative staining; 20 µl of cell suspension was directly pipetted onto a TEM square mesh copper grid and stained with 20 µl of 1 % phosphotungstic acid (PTA) for 30 s. Excess liquid was removed using filter paper and grids were allowed to air-dry prior to visualisation on the JEOL JEM 1010 Transmission Electron Microscope. Elemental analyses were performed using a scanning transmission electron microscope (sTEM; FEI Titan Thermis 60–300, Thermo Scientific) equipped with ChemiStage, with energy dispersive X-ray spectroscopy (EDS) mapping in sTEM mode, with an accelerating voltage of 60 kV. Approximately 20 µl of cell suspension were directly pipetted onto a TEM square mesh copper grid. Excess liquid was removed using filter paper and grids were air-dried prior to visualization. Cells of strain Bs02^T^ show the double layered cell envelope characteristic of Gram-negative bacteria (Fig. 1a, b) and bear a single polar flagellum made visible in TEM images by negative staining (Fig. 1c). Energy dispersive X-ray analysis reveals spots of high phosphate concentration near the cell tips, indicating the presence of polyphosphate storage granules (Fig. 1d, e).

**Fig. 1.**
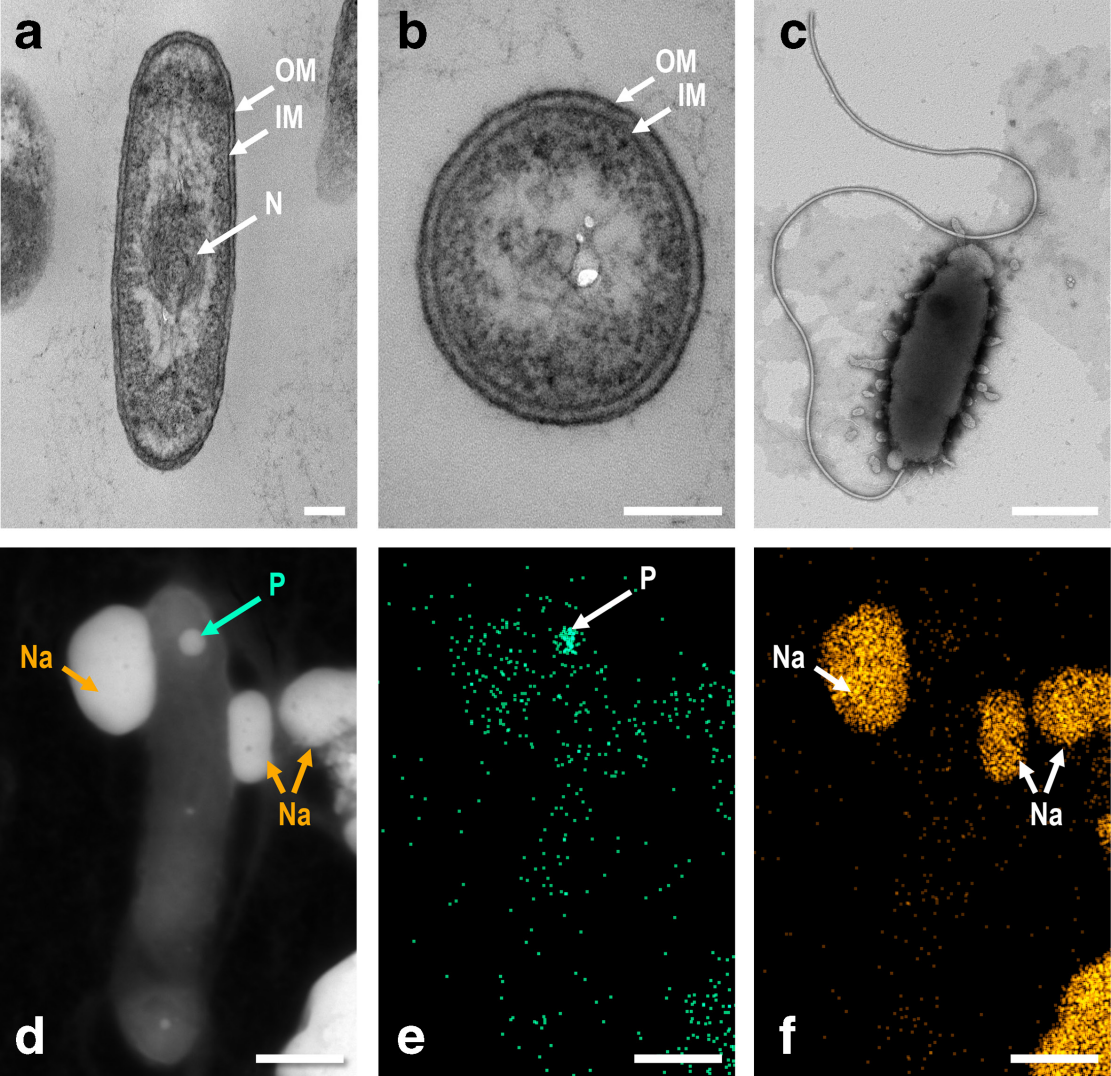
Cells of *Teredinibacter waterburyi* Bs02^T^ viewed by transmission electron microscopy. (a) Longitudinal and (b) transverse sections showing the nucleoid (N), outer membrane (OM), inner membrane (IM). (c) Negatively-stained cell showing the presence of a single polar flagellum. (d) Scanning transmission electron microscope (sTEM) image of a whole mounted cell of *T. waterburyi* Bs02^T^, (e, f) the same cell imaged by energy dispersive X-ray spectroscopy (EDS) analysis, showing (e) phosphorus and (f) sodium distribution. P, putative polyphosphate granule within the cell. Na, residual sodium precipitated from the medium. Cells were grown to mid-exponential phase in liquid SBM with 0.5 % galactose and 0.025 % NH_4_Cl at 20 °C. Bars, 100 nm (a and b), 500 nm (C), 500 nm (d–f).

Cellular fatty acid proﬁles were determined commercially using the MIDI Sherlock Microbial Identification System using cells grown on SBM agar plates supplemented with 0.5 % galactose and incubated at 20 °C for approximately one week prior to FAME analysis. The major fatty acids for strain Bs02^T^ are summed feature 5 (C_18 : 0_ ante and/or C_18 : 2_ ω6,9*c*; 59.0 %), C_16 : 0_ (13.4 %) and C_18 : 1_ ω9*c* (10.3 %) (Table S1, available with the online version of this article).

For phylogenetic analyses, cells were grown to mid-exponential phase in liquid SBM supplemented with 0.5 % galactose and pelleted by centrifugation for 10 min at 8000 *g*. Genomic DNA was extracted using the Qiagen DNeasy Blood and Tissue Kit following the manufacturer’s recommended protocol for cultured cells. Near-full length of the 16S rRNA gene was determined as described by O’Connor *et al.* [10]. Amplified products were visualised by gel electrophoresis and amplicons were cleaned and concentrated using the QIAquick PCR Purification Kit (Qiagen) following manufacturer’s protocol. Resulting products were sequenced on a 3730 XL DNA Analyzer (Life Technologies) using the Big Dye Terminator 3.1 Cycle Sequencing Kit (Life Technologies). The complete genome sequence of strain Bs02^T^ was determined using the PacBio Model RSII platform with SMRT chemistry. The sequence was assembled using HGAP version 2. A total of 6 contigs were assembled with an N50 contig length of 2 857 755 bp, which is also the length of longest contig in the assembly. The assembled genome is composed of 4 038 702 bp with an average coverage of 83× and a DNA G+C content of 47.8 mol%.

Phylogenetic analysis: The partial 16S rRNA gene sequence of strain Bs02^T^ (1420 nucleotide positions; GenBank accession MK938300) was aligned with sequences from 36 reference taxa (Figs 2 and S1) using mafft version 7 with the Q-INS-i setting [11] and trimmed to a final length of 1373 nucleotide positions. Phylogenetic analysis was performed using Bayesian inference (MrBayes version 3.2.6) [12]. Briefly, MCMC chains was set to 4 million with subsampling every 2000 generations utilizing the GTR+I+Γ nucleotide substitution model, with the first 20 % of the results discarded as the analytical burn-in. In our analysis, phylogenetic relationships among examined species within the family *Cellvibrionaceae* are poorly resolved based on analyses of 16S rRNA gene sequences alone (Figs 2 and S1), with most nodes, including those associating strain Bs02^T^ with previously named taxa, displaying posterior probabilities below commonly accepted thresholds for significance. However, within this family, approaches based on multiple protein-coding loci have been shown to produce more robust tree topologies and superior recovery of natural phylogenetic groups than those based on 16S rRNA gene sequences alone [13]. For this reason, we performed phylogenetic analyses using 37 concatenated housekeeping genes extracted from 43 available genome sequences representing members of the order *Cellvibrionales* and nine representing related reference taxa (Table S2). After manually downloading genome sequences, a custom script (see online supplement) was used to search and align the sequences using PhyloSift [14]. Phylogenetic trees were inferred based on these nucleotide sequences using RAxML version 7.04 to generate maximum likelihood trees [15]. The resulting consensus tree shows strong support for the inclusion of strain Bs02^T^ within a clade containing cellulolytic shipworm gill endosymbionts Bs08, Bs12, Bsc2 and *T. turnerae *, resolving this clade from taxa with very similar 16S rRNA sequences such as *Agarilytica rhodophyticola* 017^T^ and *Simiduia agarivorans* SA1^T^, which are free-living non-cellulolytic bacteria (Figs 3 and S2). See Table 1 for characteristics of these related bacteria.

**Fig. 2.**
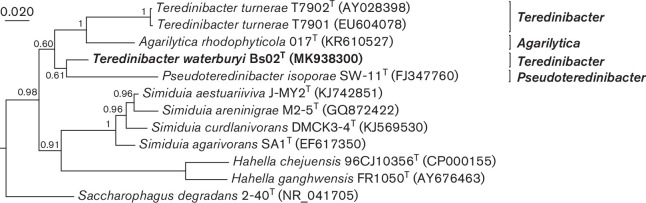
Phylogram depicting inferred relationships among *Teredinibacter waterburyi* Bs02^T^ and related bacteria based on 16S rRNA sequences. The tree presented is a subtree excerpted from the tree shown in Figure S1, inferred using 1373 nucleotide positions employing GTR+I+Γ as the substitution model in MrBayes version 3.2.6. Chain length was set to 4 million, subsampling every 2000 generations and discarding the first 20 % of the analytical results as burn-in. Posterior probability values are indicated for each node. The scale bar represents nucleotide substitution rate per site.

**Fig. 3.**
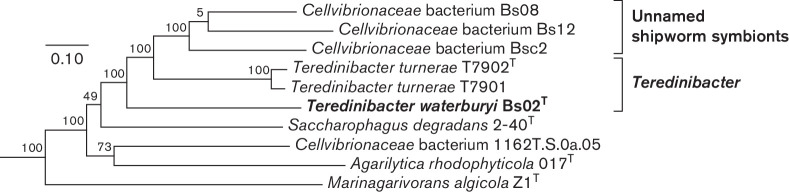
Phylogram depicting inferred relationships among *Teredinibacter waterburyi* Bs02^T^ and related bacteria based on concatenated nucleotide sequences of 37 conserved proteins. The tree presented is a subtree excerpted from the tree shown in Figure S2. PhyloSift was used to automatically mine, extract and align the a core set of 37 conserved protein-coding nucleotide sequences from whole genome sequences listed in Table S2. The maximum likelihood tree was constructed using RaxML version 7.04. Bootstrap proportions (100 replicates) are indicated for each node. The scale bar represents substitution rate per site.

**Table 1.** Characteristics of *Teredinibacter waterburyi* Bs02^T^ and other related bacteria Bacteria: 1, *Teredinibacter waterburyi* Bs02^T^ (data from this study); 2, *Teredinibacter turnerae* T7902^T^ (Distel *et al.* [1]); 3, *Agarilytica rhodophyticola* 017^T^ (Ling *et al.* [21]); 4, *Saccharophagus degradans* 2-40^T^ (Ekborg *et al.* [22]); 5, *Simiduia agarivorans* SA1^T^ (Shieh *et al.* [23]) ; *Pseudoteredinibacter isoporae* SW-11^T^ (Chen *et al.* [24]). nr, not reported.

Characteristic	1	2	3	4	5	6
Cell shape	Rods	Rods	Rods	Pleomorphic rods	Rods	Rods
Cell length (µm)	2–5	3–6	1.7–5.7	1–20	2–5	0.7–1.2
Marine salt (Mg and Ca) requirement for growth	−	+	nr	+	nr	nr
Vitamin requirement	−	−	nr	B cofactors stimulatory	nr	nr
Hydrolysis of cellulose	+	+	−	+	−	−
Hydrolysis of agar	−	−	+	+	+	−
Nitrogen fixation	−	+	−	−	−	−
Habitat	Endosymbiont of the shipworm *Bankia setacea*	Widely occurring endosymbiont of various shipworm species	Free-living, associated with the marine red alga *Glacilaria blodgettii*	Free-living, associated with salt marsh grass *Spartina alterniflora*	Free-living marine bacterium	Free-living, associated with coral *Isopora palifera*
Genome size (Mbp)	4.03*	5.38*	6.87*	5.05	4.31	nr
G+C content (mol%)	47.8	50.8	40.2	45.8	55.6	51.6

*Genome not closed

While phylogenetic analyses support a single clade containing strain Bs02^T^ and *T. turnerae* T7902^T^, pairwise 16S rRNA gene sequence identity (93.7 %) and whole genome ANI (70.0 %) [16, 17] indicate levels of identity far lower than the thresholds (99 and 95 % respectively) previously proposed to justify placement of strains within a single bacterial species [18, 19]. However, the pairwise percentage of conserved proteins (POCP) estimated for the genomes of strain Bs02^T^ and *T. turnerae* strain T7902^T^ is 57 %, falling within the 50 % threshold proposed for inclusion of bacterial strains within a single bacterial genus [20]. In contrast, the pairwise POCP values for *A. rhodophyticola* 017^T^ or *S. agarivorans* SA1^T^ and strain Bs02^T^ are estimated to be 44 and 43 %, respectively, falling far short of that threshold. Based on these findings, we propose the species *Teredinibacter waterburyi* sp. nov. for this new strain.

## Emended description of the genus *Teredinibacter* Distel *et al*. 2002

*Teredinibacter* [Te.re.di.ni.bac′ter. N.L. fem. pl. n. *Teredinidae* a family of wood-boring bivalve molluscs (shipworms); N.L. masc. n. *bacter*, equivalent of *bacterium*, a staff or rod; N.L. masc. n. *Teredinibacter* a rod isolated from members of the family *Teredinidae*].

Cells are rigid, Gram-negative, rod-shaped, 0.4–0.6 µm wide and 2–6 µm long during exponential-phase growth on SBM medium with Sigmacell cellulose as a carbon source. When grown on SBM agar plates supplemented with Sigmacell cellulose, forms colonies that are initially raised, shiny, colourless and uniformly circular. Subsequently, growth occurs beneath the agar surface forming inverted dome-shaped colonies. Mature colonies may be clear, white, yellow or brown. A halo of clearing may be observed surrounding mature colonies due to cellulose hydrolysis when grown on SBM agar plates supplemented with Sigmacell cellulose. Division is by binary fission in a single plane. Motility is by means of a single polar flagellum. In stationary-phase cultures, cells often become pleomorphic, appearing as elongate spirals or rods. Growth is aerobic or microaerobic, mesophilic and heterotrophic. The complex terrestrial plant polysaccharides cellulose and hemicellulose, as well as simple sugars support growth. Phylogenetic analysis of 16S rRNA genes place the genus within the order *Cellvibrionales* (* Gammaproteobacteria *). The type species is *Teredinibacter turnerae.*

## Description of *Teredinibacter waterburyi* sp. nov.

*Teredininbacter waterburyi* (wa.ter.bu′ry.i N.L. gen. n. *waterburyi*, named in honour of the microbiologist Dr. John Waterbury who first isolated symbionts from teredinid bivalves).

In addition to the characteristics of the genus, cells are 2–5 µm long and 0.4–0.6 µm wide when observed during the exponential phase of growth in liquid culture. Requires a source of combined nitrogen such as NH_4_Cl or nitrate for growth. Colonies are minute (typically less than 0.5 mm), translucent, white to beige in colour, uniformly circular and produce clearings due to hydrolysis of cellulose. The pH, temperature, and salinity range for growth was approximately pH 6.5–8.5, 10–30 °C, and 0.0–0.8 M NaCl, respectively; with the optimum growth recorded at pH 8.0, 20 °C, and 0.5 M NaCl. The major fatty acids are summed feature 5 (C_18 : 0_ ante and/or C_18 : 2_ω6,9*c*), C_16 : 0_ and C_18 : 1_ω9*c*. The genome is estimated to be 4.03 Mbp with a G+C content of 47.8 mol%. The type strain, Bs02^T^, has been deposited to the American Type Culture Collection (ATCC), and Korean Collection for Type Cultures (KCTC) as ATCC TSD-120^T^ and KCTC 62963^T^, respectively. The GenBank/EMBL/DDBJ accession number for the 16S rRNA gene is MK938300. The draft genome sequence was deposited under IMG Genome ID 2781125611.

## Supplementary Data

Supplementary material 1
