# Supplementary material for: Teredinibacter waterburyi sp. nov., a marine, cellulolytic endosymbiotic bacterium isolated from the gills of the wood-boring mollusc Bankia setacea (Bivalvia: Teredinidae) and emended description of the genus Teredinibacter
Source: Int J Syst Evol Microbiol. 2020 Feb 20;70(4):2388–94. doi: 10.1099/ijsem.0.004049 (PMC7395619; doi:10.1099/ijsem.0.004049)
Supplement: Supplementary material 1 [file ijsem-70-2388-s001.pdf]

Supplemental Figures and Tables

Teredinibacter waterburyi sp. nov., a marine, cellulolytic endosymbiotic bacterium isolated from the gills of the wood-boring mollusc *Bankia setacea* (Bivalvia: Teredinidae), and emended description of the genus *Teredinibacter*

Marvin A. Altamia, J. Reuben Shipway, David P. Stein, Meghan A. Betcher, Jennifer M. Fung, Guillaume Jospin, Jonathan A. Eisen, Margo G. Haygood, Daniel L. Distel

International Journal of Systematic and Evolutionary Microbiology

Corresponding author: Daniel L. Distel
Ocean Genome Legacy Center
Northeastern University
430 Nahant Road
Nahant, MA, USA
email: d.distel@neu.edu

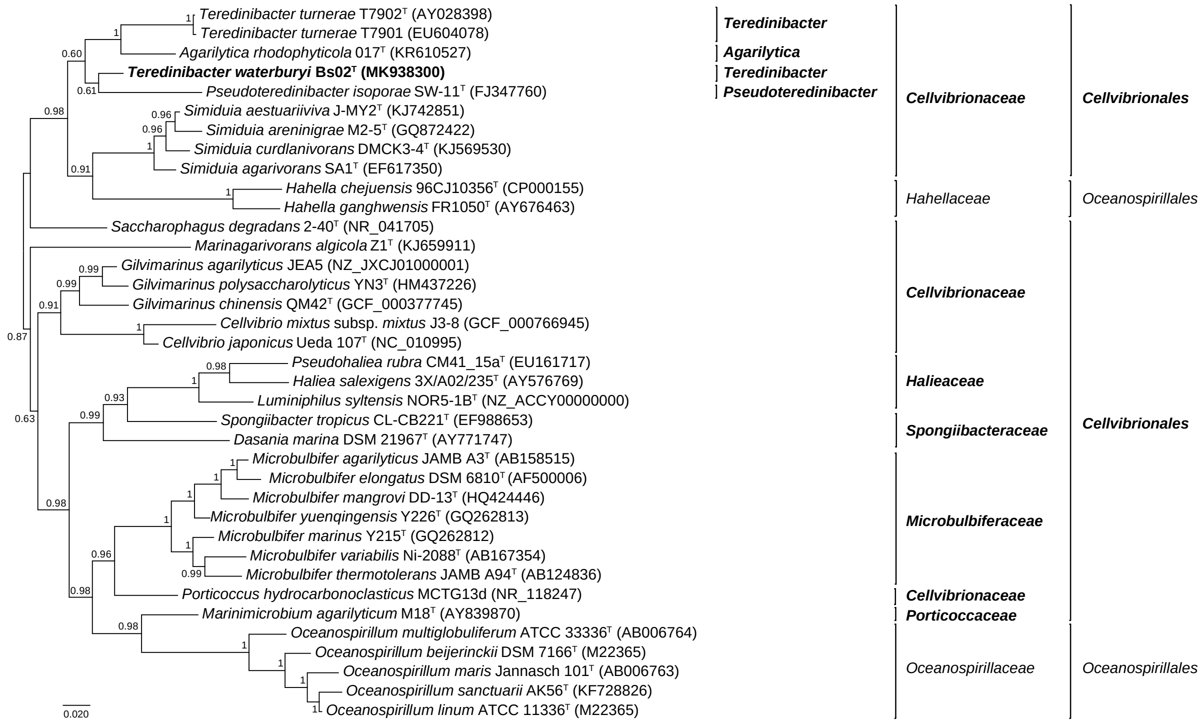

Figure S1. Phylogram depicting inferred relationships among *Teredinibacter waterburyi* Bs02^{T} and related bacteria based on 16S rRNA sequences. The Bayesian tree was constructed using 1,373 nucleotide positions employing GTR + I + Γ as the substitution model in MrBayes version 3.2.6. Chain length was set to 5 million, subsampling every 2,000 generations and discarding the first 20% of the analytical results as burn-in. Posterior probability values are indicated for each node. The scale bar represents nucleotide substitution rate per site. A subtree excepted from this tree is shown in Figure 2.

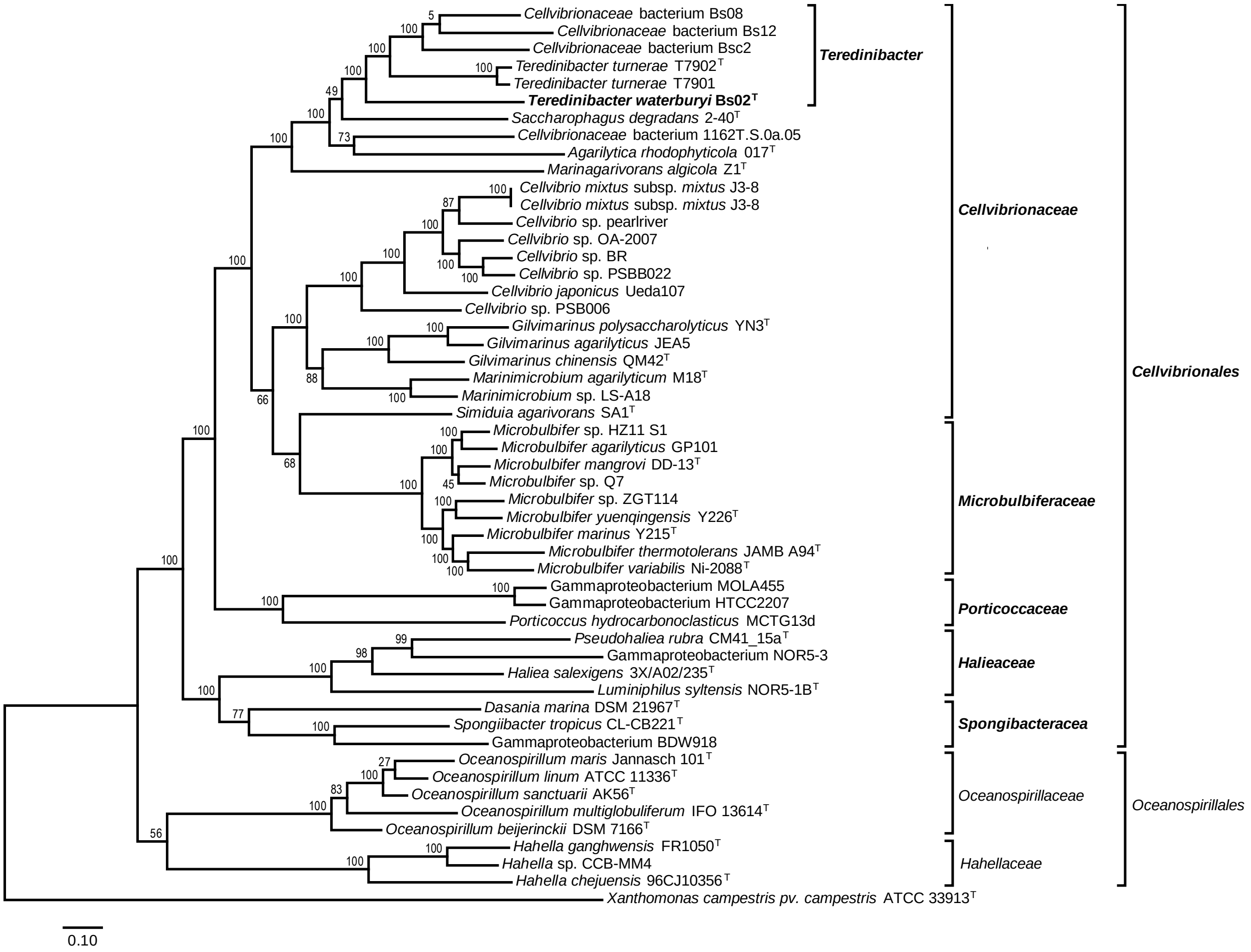

Figure S2. Phylogram depicting inferred relationships among *Teredinibacter waterburyi* Bs02T and related bacteria based on concatenated nucleotide sequences of 37 conserved proteins. PhyloSift was used to automatically mine, extract, and align the marker protein sequences from the whole genome sequences listed in Table S2. The maximum likelihood tree was constructed using RaxML version 7.04. The tree was rooted with *Xanthomonas campestris*. Bootstrap proportions (100 replicates) are indicated for each node. The scale bar represents substitution rate per site. A subtree excepted from this tree is shown on Figure 3.

Table S1. Cellular fatty acid contents (percentage) of *T. waterburyi* Bs02^{T} related bacteria

Bacteria: 1, *Teredinibacter waterburyi* Bs02^{T} (data from this study); 2, *T. turnerae* T7902^{T} (21); 3, *Agarilytica rhodophyticola* 017^{T} (21) . For strains 2 and 3, tr denotes traces (<0.5%). The three most abundant fatty acids in each type strain are in boldface; -, not detected.

**Fatty acids**	**1**	**2**	**3**
$C_{9:0}$	0.9	-	-
$C_{10:0}$	-	6.6	6.8
$C_{11:0}$	-	tr	0.5
$C_{10:0}$ 3-OH	-	2.5	6.0
Unknown 11.799		1.5	–
$C_{12:0}$	1.1	3.3	9.0
$C_{12:1}$ 3-OH	-	0.7	–
$C_{12:0}$ 2-OH	-	–	–
$C_{12:0}$ 3-OH	-	4.2	2.2
$C_{14:0}$	1.0	7.2	–
$C_{15:0}$	-	0.8	1.2
$C_{16:0}$	**13.4**	**19.7**	**30.8**
iso-$C_{17:0}$	-	–	0.7
$C_{17:0}$	-	tr	1.0
$C_{17:0}$ 2-OH	0.6	-	-
$C_{17:1}$ ω8*c*	0.4	-	
iso-$C_{16:0}$ 3-OH	-	–	3.2
$C_{18:1}$ ω9*c*	**10.3**	-	**-**
$C_{18:1}$ ω7*c*	3.0	6.1	**23.1**
$C_{18:1}$ ω6*c*	-	**24.3**	–
$C_{18:0}$	2.3	tr	0.8
iso-$C_{19:0}$	0.4	-	-
Summed feature 2*	-	–	0.6
Summed feature 3*	7.7	**21.9**	**12.2**
Summed feature 5*	**59.0**	**-**	**-**
Total	100.0	98.7	98.1

Summed features are peaks consisting of two or more fatty acid methyl esters that that cannot be separated in MIDI system. Summed feature 2: $C_{14:0}$ 3-OH and/or iso-$C_{16:1}$; Summed feature 3: $C_{16:1}$ ω7*c* and/or iso-$C_{15:0}$ 2-OH; Summed feature 5: $C_{18:0}$ ante and/or $C_{18:2}$ ω6,9*c*.

Table S2: Whole genome sequence assemblies used to construct a protein-based phylogenetic tree.

	Species name	Whole genome assembly accession
1	*Teredinibacter waterburyi* Bs02T	IMG 2781125611
2	*Cellvibrionaceae* bacterium Bsc2	IMG 2531839719
3	*Cellvibrionaceae* bacterium Bs12	IMG 2545555829
4	*Cellvibrionaceae* bacterium Bs08	IMG 2781125612
5	*Teredinibacter turnerae* T7902T	GenBank GCA_000379165.1
6	*Teredinibacter turnerae* T7901	GenBank GCA_000023025.1
7	*Cellvibrionaceae* bacterium PMS-1162T.S.0a.05	GenBank GCA_000964245.1
8	*Agarilytica rhodophyticola* 017T	GenBank GCA_002157225.1
9	*Saccharophagus degradans* 2-40T	GenBank GCA_000013665.1
10	*Marinagarivorans algicola* Z1T	GenBank GCA_001292705.1
11	*Cellvibrio* sp. PSBB023	GenBank GCA_002007605.1
12	*Cellvibrio mixtus* PSBB022	GenBank GCA_002268635.1
13	*Cellvibrio* sp. BR	GenBank GCA_000263355.1
14	*Cellvibrio* sp. OA-2007	GenBank GCA_000953825.1
15	*Cellvibrio* sp. pealriver	GenBank GCA_001183545.1
16	*Cellvibrio mixtus* subsp. *mixtus* J3-8	GenBank GCA_000766945.1
17	*Cellvibrio japonicus* Ueda107	GenBank GCA_000019225.1
18	*Cellvibrio* sp. PSBB006	GenBank GCA_002162135.1
19	*Gilvimarinus agarilyticus* JEA5	GenBank GCA_000832015.1

20	*Gilvimarinus polysaccharolyticus* YN3^T	GenBank GCA_001187555.1
21	*Gilvimarinus chinensis* QM42^T	GenBank GCA_000377745.1
22	*Marinimicrobium* sp. LS-A18	GenBank GCA_000463525.1
23	*Marinimicrobium agarilyticum* M18^T	GenBank GCA_000423345.1
24	*Microbulbifer thermotolerans* JAMB A94^T	GenBank GCA_900112305.1
25	*Microbulbifer variabilis* Ni-2088^T	GenBank GCA_000380565.1
26	*Microbulbifer marinus* Y215^T	GenBank GCA_900107725.1
27	*Microbulbifer* sp. ZGT114	GenBank GCA_001507605.1
28	*Microbulbifer yueqingensis* Y226^T	GenBank GCA_900100355.1
29	*Microbulbifer agarilyticus* GP101	GenBank GCA_001999945.1
30	*Microbulbifer* sp. HZ11 S1	GenBank GCA_000708675.1
31	*Microbulbifer* sp. Q7	GenBank GCA_001639145.1
32	*Microbulbifer mangrovi* DD-13^T	GenBank GCA_002009015.1
33	*Simiduia agarivorans* SA1^T	GenBank GCA_000420285.1
34	Gammaproteobacterium MOLA455	GenBank GCA_000511875.1
35	Gammaproteobacterium HTCC2207	GenBank GCA_000153445.1
36	*Porticoccus hydrocarbonoclasticus* MCTG13d	GenBank GCA_000744735.1
37	*Pseudohaliea rubra* CM41_15a^T	GenBank GCA_000764025.1
38	Gammaproteobacterium NOR5-3	GenBank GCA_000158155.1
39	*Haliea salexigens* 3X/A02/235^T	GenBank GCA_000423125.1
40	*Luminiphilus syltensis* NOR5-1B^T	GenBank GCA_000158175.1
41	Gammaproteobacterium BDW918	GenBank GCA_000259575.1

42	*Spongiibacter tropicus* CL-CB221^T	GenBank GCA_000420325.1
43	*Dasania marina* DSM 21967^T	GenBank GCA_000373485.1
44	*Oceanospirillum sanctuarii* AK56^T	GenBank GCA_002150805.1
45	*Oceanospirillum linum* ATCC 11336^T	GenBank GCA_001995095.2
46	*Oceanospirillum maris* Jannasch 101^T	GenBank GCA_000422865.1
47	*Oceanospirillum beijerinckii* DSM 7166^T	GenBank GCA_000422425.1
48	*Oceanospirillum multiglobuliferum* IFO 13614^T	GenBank GCA_900167095.1
49	*Hahella ganghwensis* FR1050^T	GenBank GCA_000376785.1
50	*Hahella chejuensis* 96CJ10356^T	GenBank GCA_000012985.1
51	*Hahella* sp. CCB-MM4	GenBank GCA_002260525.1
52	*Xanthomonas campestris* pv. *campestris* ATCC	GenBank GCA_000007145.1

Supplemental scripts

run_raxml.sh

```
#!/bin/bash


mkdir raxml_Bankia_16s

cd raxml_Bankia_16s


raxmlHPC-PTHREADS -m GTRGAMMA -n Bankia_setacea_Isolates.2.16S -x 47 -T 15 -f a -p 47 -N 1000 -s ../Bankia_setacea_Isolates.2.16S.fa


cd ..

mkdir raxml_Bankia_concat_nucl

cd raxml_Bankia_concat_nucl

raxmlHPC-PTHREADS -m GTRGAMMA -n Bankia_setacea_Isolates.2.concat.nucl -x 47 -p 47 -T 15 -f a -N 1000 -s ../Bankia_setacea_Isolates.2.concat.nucl.fa


cd ..

mkdir raxml_Bankia_AA

cd raxml_Bankia_AA


raxmlHPC-PTHREADS -m PROTGAMMABLOSUM62 -n Bankia_setacea_Isolates.2.AA -p 47 -x 47 -T 15 -f a -N 1000
```
